# Mapping, Structure and Modulation of PPI

**DOI:** 10.3389/fchem.2021.718405

**Published:** 2021-10-07

**Authors:** Elisa Martino, Sara Chiarugi, Francesco Margheriti, Gianpiero Garau

**Affiliations:** ^1^ Laboratorio NEST, Scuola Normale Superiore, Pisa, Italy; ^2^ BioStructures Lab, Istituto Italiano di Tecnologia (IIT@NEST), Pisa, Italy

**Keywords:** PPI in disease1, target structure2, modulation strategies3, protein-protein4, ligand interaction5

## Abstract

Because of the key relevance of protein–protein interactions (PPI) in diseases, the modulation of protein-protein complexes is of relevant clinical significance. The successful design of binding compounds modulating PPI requires a detailed knowledge of the involved protein-protein system at molecular level, and investigation of the structural motifs that drive the association of the proteins at the recognition interface. These elements represent hot spots of the protein binding free energy, define the complex lifetime and possible modulation strategies. Here, we review the advanced technologies used to map the PPI involved in human diseases, to investigate the structure-function features of protein complexes, and to discover effective ligands that modulate the PPI for therapeutic intervention.

## Introduction

Many relevant human pathologies, including cancer, neurodegenerative and infection diseases, are the result of abnormal protein–protein interactions (PPI) that alter the mechanism of molecular recognition and the affinity of binding partners under a given set of conditions (Vidal et al., 2011; Maniaci and Ciulli, 2019; Lu et al., 2020; Huttlin et al., 2021). Protein recognition relies on few surface residues (named interaction “hot spots”) and on the presence of water molecules at the binding interface, which play a key role in the interaction between a protein and its binding site (Ringe, 1995; Rajamani et al., 2004; Ran and Gestwicki, 2018). The interactions are driven by the concentration of the singular associating components and by the free energy of the complex, relative to alternative states (Nooren and Thornton, 2003; Maurer et al., 2018).

PPI group dynamic systems where interacting proteins are continuously involved in a very wide range of activities and signaling processes of protein folding, association, transport, and degradation. During the life cycle of a protein, its chemical interactions are indeed more important than hard-core repulsions under physiological conditions in cell (Speer et al., 2021). Hence, the association kinetics and dissociation kinetics are key parameters for the PPI. Permanent PPI have a relatively long half-life and generally account obligate complexes, while transient protein complexes form and break down transiently *in vivo* (Perkins et al., 2010). Stable PPI are usually mediated by reciprocal recognition interfaces of the proteins, while transient PPI are frequently formed between globular domains and short linear peptide motifs or small structural epitopes.

Most PPI take on the appearance of oligomers that function only after the association of multiple copies of the chains, as for key membrane protein targets (Magotti et al., 2015). PPI of oligomeric proteins show often exceptional selectivity to perform their functional role, even in the presence of closely related proteins (Hochberg et al., 2018).

As PPI do not have natural small-molecule partners that can be used as starting compound ligand hits (Wells and McClendon, 2007), the successful design of modulators affecting PPI is an enormous challenge, and in many cases, it is strongly supported by a detailed knowledge of the specific protein-protein system at molecular and structural level (Scott et al., 2016). Here we review recent developments in identification and validation of protein-protein complexes, and in structural biophysics approaches used to discover effective modulators for therapeutic intervention.

## Mapping OF PPI IN Diseases

Interactome maps of high complexity are becoming increasingly available to decipher disease-specific protein associations and characterize the effects of splicing and genetic variation on these systems (Vidal et al., 2011; Wang et al., 2012; Bludau and Aebersold, 2020; Huttlin et al., 2021). Recent works show that compared to germline variants identified in healthy participants, disease-associated alleles commonly alter specific PPI rather than affecting folding or stability of single proteins. For example, PPI-perturbing mutations are significantly associated with poor survival rate in cancer patients, while mutations in the gene alone did not typically correlate with patient survival (Cheng et al., 2021). Among the techniques used to merge validated PPI with diseases, the network maps of correlated mRNA expression and the *in-silico* genome analysis are powerful methods for discriminating cell states and disease outcomes (von Mering et al., 2002; Cheng et al., 2021). Coupled with the analysis of physical protein-protein interactome, the investigation of network maps can also generate lists of genes potentially enriched for new candidate disease genes or modifier genes of known disease genes (Vidal et al., 2011; Bludau and Aebersold, 2020; Huttlin et al., 2021).

Other than genomic-based approaches with interactome information, large-scale proteomics methods have been used in recent years to identify PPI involved in human diseases, and to link genetics and physiology observations (Luck et al., 2020; Huttlin et al., 2021). PPI are incredibly diverse, and a proteome-scale map provides a global view of organization of cell processes and protein functions (Bludau and Aebersold, 2020). In addition, the proteomic approach can unveil the biological and pathological mechanisms of associated diseases, as well as explore key aspects of possible modulation strategies for therapeutic intervention. (Rual et al., 2005; Huttlin et al., 2015; Li et al., 2017; Huttlin et al., 2021).

Most of our knowledge about PPI networks in all organisms are largely derived from binary interaction mapping by two-hybrid technologies and protein chip technologies, which continue to have a dominant role in the assessment of protein interactomes and exploitation of the PPI therapeutic potential (Suter et al., 2008). The resulting map of binary interactions (interactome) covers now more than ∼64.000 PPI in human (www.interactome-atlas.org). These technologies identify efficiently direct physical interactions between two proteins. However, they require proteins to be expressed at non-endogenous levels and often are not able to capture all interactions involving intermediary or scaffold proteins. Another high-throughput proteome-scale mapping uses epitope tags fused to bait proteins (a known protein that is used to covalently label its partner protein). Protein associated with the bait are then identified and characterized using affinity purification or co-fractionation followed by mass spectrometry (Cafarelli et al., 2017). Recently, to identify specifically PPI involving membrane proteins (30% of human proteins and more than 60% of current drug targets) an approach that uses a small protein-tag (Pup) fused to proteins that interact with a PafA-fused bait have demonstrated to allow identification of transient and weak interactions by mass spectrometry (Liu et al., 2018).

Although the characterization of PPI by genetic and proteomics approaches is often sufficient to confirm the interactions, direct visualization of these interactions at cellular level provides an additional level of validation and characterization. Among different techniques, we mention the approach of fluorescence resonance energy transfer (FRET), which is often used as intracellular assay for PPI and their dynamics in cells (Weiss, 1999; Gul and Hadian, 2014). Non-radiative energy is transferred from a donor fluorophore coupled to a protein, to an acceptor fluorophore coupled to another protein, if two potential partners are close enough (within ∼10 nm). The method can be applied both *in vivo* and *in vitro* with resolution at the nanometer scale. Recent advances in the field include 1) the development of FRET-based high-throughput screenings in living cells (Stroik et al., 2018); 2) a photo-switching FRET system based on a donor molecule having photo-switching properties, which are slower in the presence or in the absence of an acceptor (Rainey and Patterson, 2019); 3) a FRET system consisting of a protein target incorporated with a fluorescent amino acid and a yellow fluorescent protein (YFP) fusion protein (Park et al., 2019).

## PPI Resources

The results of gene screenings and proteomics are reported in different online databases, including interactions across thousands of published studies and experimental techniques. The classification of PPI is made on the base of different parameters, including protein domains, type of interactions, identification of involved species and detection methods (Stacey et al., 2018).

Among these, 1) BIOPLEX (http://thebiogrid.org) is an interactome dataset that includes nowadays nearly 120,000 interactions among nearly 15,000 proteins, and different cell-line-specific interaction networks obtained from proteomics approaches. It is the most comprehensive experimentally derived model of the human interactome to date. It comprises 5,522 baits in human colon cancer cell lines (HCT116) (Huttlin et al., 2021). Other databases include: 2) HuRI (www.interactome-atlas.org) is a server that integrates human data of genome, transcriptome and proteome, enabling cellular function to be studied within most physiological or pathological cellular contexts, with more than 64,000 PPI (Luck et al., 2020); 3) STRING (https://string-db.org) is a database of known and predicted PPI. The interactions include direct (physical) and indirect (functional) associations, from knowledge transfer between organisms and other databases; 4) IntAct (https://www.ebi.ac.uk/intact) of PPIs derived from individual user submissions and literature observations; 5) Interactome3D (http://interactome3d.irbbarcelona.org) a web service for the structural annotation of PPI networks; 6) APID (http://cicblade.dep.usal.es:8080/APID/init.action) a protein interactomes data server; 7) Pathway Commons that merge information of both pathways and interactions (http://www.pathwaycommons.org); 8) BioGRID (https://thebiogrid.org) is a biomedical interaction repository of proteins, genetic and chemical interactions, with data compiled through comprehensive curation efforts; 9) I2D is a database that aims to facilitate experimentation using experimental and predicted PPI for five model organisms and human (http://ophid.utoronto.ca/iid).

Structure coordinates of validated PPI can be extracted directly from protein complexes obtained from experiments of X-ray crystallography (X-ray diffraction, XRD), single particle cryo-electron microscopy (cryoEM) and other methodologies, contained within the (10) Protein Data Bank (PDB) (http://www.rcsb.org/pdb) (Rose et al., 2017). The 11) ProtCID server (http://dunbrack2.fccc.edu/ProtCiD/Search/Uniprots.aspx) is a data resource for structural information on protein interactions. It provides clusters of interfaces of full-length protein chains and functional domains as a means of identifying biological assemblies, showing information on four types of interactions: protein–protein interactions at the chain level, protein–protein interactions at the domain level, domain–peptide interactions, and also the interactions of domains with nucleic acids and ligands. Within the ProtCID server, the PDBfam database contains 8636 protein domain families (Pfams) present in the PDB (Xu and Dunbrack, 2020).

When the experimentally determined structures of involved proteins are available, long-timescale molecular dynamics simulations using enhanced sampling can virtually illustrate the spontaneous association and dissociation processes, extracting mechanistic insights into the PPI dynamics (Pan et al., 2019). As structures describing PPI are still relatively underrepresented in the PDB, potent suites of homology search, template-based modeling, structure prediction and macromolecular docking for robust and fast protein–protein docking are available to estimate and visualize hypothetical coordinate interactions. Among these, we mention the servers 12) Hdock (http://hdock.phys.hust.edu.cn) (Yan et al., 2020; Soni and Madhusudhan, 2017) and 13) ROSETTA Commons (https://www.rosettacommons.org) (Lyskov and Gray, 2008). While structural predictions *in silico* are relatively easy to obtain, the resulting virtual structure representations require always accurate experimental validation.

## PPI Structures

The most important lesson learned from the successful design of compounds against PPI is the value of quality structural information describing the protein interaction and involved binding motifs (Corbi-Verge and Kim, 2016). Expanding knowledge of a specific protein-protein complex at the molecular level provides strong insights also into the dynamics of the protein association events and the prioritization of actionable biomarkers.

XRD has a dominating role in solving biomolecular structures at atomic resolution. The volumetric data obtained by this technique (electron density map) describes the position of atoms of the protein-protein complex of interest. When the structure of the individual protein partners is solved, the structure of the entire complex can be uncovered and modelled using a combination of complementary approaches that include mutagenesis, chemical cross-linking and analytical ultracentrifugation using computational methods (Cicaloni et al., 2019). Solving the crystal structure of the protein complex allows determining the interaction “hotspots” to atomic resolution, directly. It allows for an assessment of customized compound library strategies and can make virtual screening of compound libraries efficacious (Table 1). A recent development in the field is the potential to perform a rational design of PPI-modulating compounds with the aim to develop PPI specific stabilizers, starting from weak and promiscuous ligands seen in complex crystal structures (Sijbesma et al., 2020).

**TABLE 1:** Biophysical methods for ligand discovery targeting PPI.

Technique	Acronyms	Method	Pros	Cons
Fluorescence Polarization	FP	It uses a fluorescent ligand that changes polarization when interacts with/dissociates from the protein target. It is carried out in a competitive inhibition mode, with a labeled truncated protein containing “hot-spots”.	Simple; low cost; low volumes; suitable for HTS	Introduction of fluorophore tags; non-native binding properties
Amplified Luminescent Proximity Homogeneous Assay Screen	AlphaScreen	Chemiluminescent technology that uses a donor bead (DB) and an acceptor bead (AB), with emission signal at interaction (<200 nm). It is carried out in a competitive inhibition mode	Sensitive; label-free proteins; suitable for HTS; relatively expensive	It needs a specialized plate reader; it can generate false positive
Fluorescence Resonance Energy Transfer	FRET	It uses nonradiative energy transfer between an excited probe (donor), and an accepted probe (acceptor) at interaction (<10 nm). The presence of a PPI dissociating ligand alters the emission wavelength. Time-resolved FRET introduces lanthanide ions as donor to limit signal contaminations	Sensitive; low-cost; low volumes; used with a range of protein sizes	Each interacting protein partner need to be fused with a fluorescent protein; findings can be altered by fluorescence ligand interference
Differential Scanning Fluorimetry (Thermal Shift Assay)	DSF (TSA)	It uses the biding of hydrophobic fluorescent dye to hydrophobic regions of protein targets. The presence of a ligand stabilizing (destabilizing) the PPI increases (decreases) the melting temperature	Simple; Low cost; label-free proteins; immobilization free; suitable for HTS; it can be done in common real-time PCR machines	It is incompatible with low solubility compounds
Nuclear Magnetic Resonance	NMR	NMR experiments identify binding events either by looking at the resonance signals of the ligand or the protein. It can detect non-specific ligand binding. Methods include approaches of Water-LOGSI, Saturation Transfer Difference, Spin Labelling, Inverse NOE pumping	Very sensitive and valuable; label free; immobilization free; it provides epitope mapping, binding affinity (from pM to mM) kinetics and thermodynamics	It is incompatible with low solubility of compounds and targets; it requires high protein concentration; 2D NMR mapping requires labeled proteins; expensive equipment
Surface Plasmon Resonance	SPR	Processes that alter the local refractive index (ligand or protein adsorption) onto the biosensor layer (with an immobilized partner) can be monitored in a surface sensitive fashion by recording the shift of the resonance minimum	Very sensitive; label free; it provides affinity (from nM to mM) kinetics and thermodynamics of protein-protein association/dissociation and ligand binding; a gold standard of PPI, suitable for HTS	It requires immobilization of a binding partner; generally, it requires a positive reference ligand to limit false negatives
Isothermal Titration Calorimetry	ITC	It measures directly the enthalpic energy contribution associated with the binding reaction of two components, and the associated interaction free energy by titration	Sensitive; label free; immobilization free; it directly measures all thermodynamics parameters	It requires high amount of both ligand and protein; expensive equipment
Mass Spectrometry	MS	It detects ligands using irreversible binding compounds/fragments, and approaches of disulfide tethering on targets containing both native and introduced cysteine residues	Very sensitive; label free. It provides epitope mapping; suitable for HTS	It is incompatible with not irreversible ligands; expensive equipment
X-ray Crystallography (Diffraction)	XRD	The electron density map obtained from X-ray diffraction directly yields a high-resolution picture of the ligand–protein complex, providing atomic level insights into the physical chemistry of complex formation	Very powerful technique for studying and validating protein-protein/ligand interactions at atomic resolution. Complex structures can be generated very rapidly. It gives key initial components for molecular dynamics and structure- or fragment-based drug design	It needs high amount of sample and known protein crystallization conditions. Complex structures tend to be more problematic to interpret unambiguously at low-resolution (>3 Å)
Single particle Cryo-Electron Microscopy	CryoEM	The Coulomb potential map can be used to determine at near-atomic resolution the structure of biological macromolecules and large protein complexes that are not accessible to X-ray crystallographic analysis	Powerful technique for studying and validation protein-protein and protein-ligand interactions at near atomic resolution. The reconstruction of various intermediate states can help to understand the dynamics of a complex system	Sample preparation often requires a great deal of optimization. The resolution is often limited to 3–4 Å. Each data collection spans the course of several hours or days, making the throughput for cryo-EM much slower than XRD.

The concept that weak compounds with reduced molecular complexity provide efficient sampling of chemical space is known to be central in fragment-based ligand discovery, which has become a mainstream technology for the identification of efficient chemical starting hits in current drug discovery programs (O’Reilly et al., 2019). Metrics to assess the drug-like quality of a binding compound include the ligand efficiency (LE = ΔG/HA; LE values close or higher than 0.3 are desired) and the lipophilic ligand efficiency (LLE = pIC_50_–logP or logD; values > 5 are considered favorable for *in vivo* activity) (Hopkins et al., 2014). The requirement of high ligand efficiency during the evolution of PPI-modulating compounds is due to the average surface size of protein-protein contacts to modulate (∼1,500–3,000 Å^2^), with compared to the average surface size of small molecule-protein when targeting single proteins (∼300–1,000 Å^2^) (Wells and McClendon, 2007).

Most of the structures reported in the PDB archive (∼90%) have been determined using XRD, including those of protein-protein complexes. Another technology, single particle cryoEM is starting to become instrumental in structure determination of macromolecular complexes, for targets that are either very hard to produce in large quantity or almost impossible to crystallize (Cheng, 2018). The three-dimensional surface density of a protein complex is a reconstruction from a set of images (two-dimensional projections) of the assembly, imaged at various orientations, by the electron microscope. Contrary to the electron density map obtained from experiments of crystal diffraction, the image electron scattering is the result of the incident electrons interacting with the local atomic charges (Coulomb potential). Macromolecular complexes can be assessed by their overall fit to the experimental data and stereochemical information. Despite it provides results having normally lower resolution than XRD (Table 1), the resulting Coulomb potential maps can provide additional details about the electrostatic environment and charge state of atoms (Hryc et al., 2017).

Protein-protein complex formation may be eventually studied using Nuclear Magnetic Resonance (NMR) spectroscopy (Bonvin et al., 2005). The cross-saturation method in NMR has been specifically developed to identify the interfaces of large (Mr > 50,000) protein–protein complexes (Takahashi et al., 2000) (Table 1). Contrary to previous methodologies, the advanced applications of the NMR technology allow mapping structural interactions also in cells (STINT-NMR) (Burz et al., 2006). In the last years, methods of Mass Spectrometry (Walzthoeni et al., 2013), Small-Angle X-ray Scattering (Kikhney and Svergun, 2015), and 3D electron microdiffraction (Lanza et al., 2019) have been also developed to provide structural information of protein-protein complexes at low resolution.

### TECHNIQUES AND MODULATION STRATEGIES

In recent years, some PPI-modulating compounds have entered clinical studies, and few of them have been successfully approved for treatment of diseases, in particular for cancer (Ran and Gestwicki, 2018; Lu et al., 2020). The compounds can target the protein interaction interface directly or an allosteric site on one of the involved partner proteins, inhibiting or enhancing the complex function.

High-throughput screenings can yield useful starting points for chemical optimization (Higueruelo et al., 2013; Gul and Hadian, 2014). Because of the general absence of binding pockets at the interfaces of PPI, the compound library used for screening need to have a high chemical diversity and strong potential of providing very high ligand efficiency to match the protein-protein association. Effective screening techniques include bioassays of fluorescence resonance energy transfer (FRET) and fluorescence polarization (FP), amplified luminescent proximity (AlphaScreen) and thermal shift assay (TSA) (Table 1).

The rational design of small molecules to target specific PPI strongly benefits from a better understanding of how such compounds bind at the interfaces of the complex. Highly sensitive technologies, including X-ray Crystallography (XRD), surface plasmon resonance (SPR), nuclear magnetic resonance (NMR), isothermal titration calorimetry (ITC) and mass spectrometry (MS), are frequently used for this purpose (Table 1). X-ray crystallography and single particle Cryo-EM and NMR are essential in providing structural information for the mechanism of modulation of PPI and for the chemical evolution of compounds (above). The NMR spectroscopic parameters (e.g., chemical-shift changes, changes in relaxation times, changes of diffusion constants, changes of Nuclear Overhauser Effects, NOE, or exchange of saturation) may serve as a gauge for both screening and binding activity of ligands against a protein complex. Different experimental NMR approaches exist. In general, the methodology focuses on NMR signals of the ligand, and usually it utilizes the signals NOE between the ligand and the target complex. Another NMR methodology focuses on changes of the chemical-shift of the target complex upon binding of the ligand (Meyer and Peters, 2003). Specific NMR-based screenings have proven capable of overcoming some of the challenges posed by the low solubility of membrane proteins by immobilizing the reference target (technique NMR-TINS) in two compartments of a dual-cell sample holder, and simultaneously injecting mixtures of compounds (Marquardsen et al., 2006).

**FIGURE 1:**
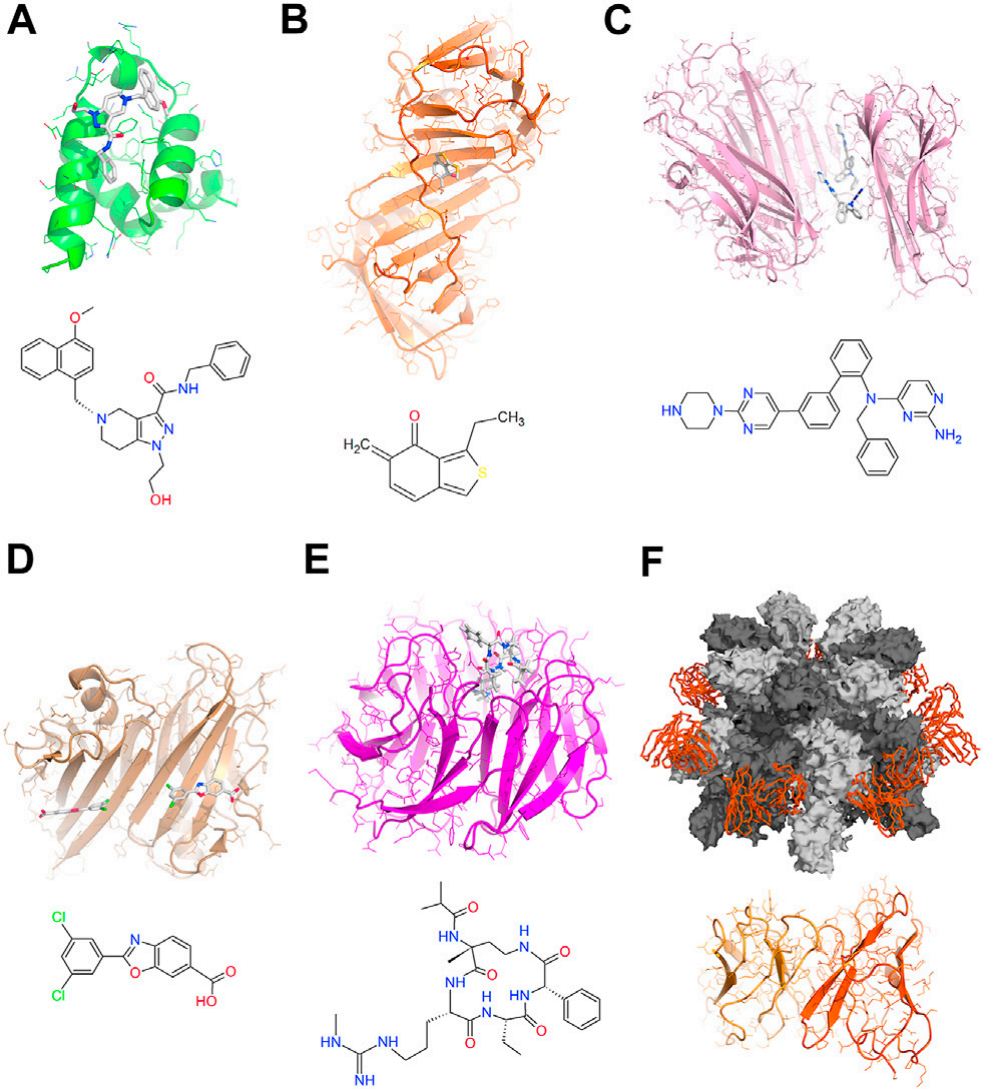
Selection of modulators targeting PPI **(A)** Structure of PEX14 bound the inhibitor [1-(2-hydroxyethyl)-5-[(4-methoxynaphthalen-1-yl)methyl]-∼(N)-(phenylmethyl)-6,7-dihydro-4∼(H)-pyrazolo (4,3-c)pyridine-3-carboxamide) that efficiently disrupts the PEX14-PEX5 interaction to treat trypanosomiases (Dawidowski et al., 2017) **(B)** Structure of human DNA polymerase processivity factor UL44 in complex with the covalent allosteric inhibitor {(5-[(dimethylamino)methylene-3-(methylthio)-6,7-dihydrobenzo (c) thiophen-4(5H)-one]} that blocks the UL44-UL54 peptide interactions to treat human cytomegalovirus infections (Chen et al., 2017) **(C)** Structure of human TNFα in complex with the inhibitor JNJ525 {(N)4-(phenylmethyl)-∼(N)4-{2-[3-(2-piperazin-1-ylpyrimidin-5-yl)phenyl]phenyl}pyrimidine-2,4-diamine} that blocks the TNF-TNFR1 signaling stabilizing a distorted TNFα complex (McMillan et al., 2021) **(D)** Structure of human Transthyretin in complex with the ligand Tafamidis [2-(3,5-dichlorophenyl)-1,3-benzoxazole-6-carboxylic acid] a potent and selective stabilizer that inhibits the amyloid cascade for the treatment of amyloid cardiomyopathy (Maurer et al., 2018) **(E)** Structure of the WD repeat domain five in complex with the macrocyclic peptidomimetic MM-589 {N-[(3R,6S,9S,12R)-6-ethyl-12-methyl-9-[3-(N′-methylcarbamimidamido)propyl]-2,5,8,11-tetraoxo-3-phenyl-1,4,7,10-tetraazacyclotetradecan-12-yl]-2-methyl propanamide} that blocks the WDR5-mixed lineage leukemia (MLL) protein-protein interaction (Karatas et al., 2017) **(F)** Structure of the anthrax toxin prepore in complex with the neutralizing Fab portion of the antibody cAb29 (Hoelzgen et al., 2021).

Specific compounds targeting PPI can be designed to inhibit/dissociate or to stabilize/enhance the protein-protein complex (Figure 1). When a ligand binds to the protein-protein interface, it can provide less or more contact sites for the two proteins, thus inhibiting/dissociating or stabilizing/enhancing the complex, respectively. Most of the small molecules that have been identified so far to modulate PPI are inhibitors or dissociating agents (Figures 1A,B). However, due to the natural driving force mediating PPI, the stabilization of protein complexes may represent a promising modulation strategy for therapeutic intervention (Figures 1C,D) (Singh et al., 2011; O’Connell et al., 2019; Sijbesma et al., 2020). When the protein-protein interface forms an appropriate binding site, developing compounds based on structural information to directly targeting this site can be very effective (Figure 1E). If the ligand binds to an allosteric/regulatory site of the protein far from the protein contact interface, it generally induces a conformation change that can reduce or enhance the affinity of the target protein to the protein partners of the PPI (Figure 1F). The modulation of allosteric/regulatory sites is known to be particularly challenging to obtain.

On the base of the chemical features, the PPI-modulating compounds can be classified into three main categories: 1) *Small molecules*. These compounds should cover generally a large surface area and make many hydrophobic contacts to affect PPI, thus can face pharmacokinetic issues. The small molecules are in principle more suitable for tight and narrow PPI interfaces (Figures 1A–D). An innovative group within these compounds is represented by heterobifunctional molecules that recruit a specific PPI target to an E3 ubiquitin ligase, resulting in the ubiquitination and degradation of the target (Bondeson et al., 2015). 2) *Structural peptides*. These compounds group peptide mimetics and synthetic peptides of structural elements (helices or strands). The development of these compounds is strongly based on PPI structural information. They work binding to a protein interface mimicking the partner protein (Figure 1E). Generally, they show high target specificity and affinity, and are preferred for PPI with large interaction surface areas (>2000 Å^2^). They are susceptible to hydrolysis and require stabilization processes (stapled peptides, foldamers, and hydrogen bond surrogates) to prolongate their half-life (Klein, 2017). Finally, 3) *Antibodies*. These potent PPI effectors can target selectively exposed cell membrane receptors. However, the incorporation of effective translational strategies from the early stages of the antibody development process is a necessity (Mohammad et al., 2009). Using single particle cryo-EM, we recently reported the first structure of the heptameric protective antigen, the central component of the anthrax toxin, in complex with a potent monoclonal antibody (cAb29) (Figure 1E). Results provide the structural basis for the antibody-based neutralization of the macro-molecular assembly responsible for the *Bacillus anthracis* lethal infection, identifying the membrane-penetrating loop of the complex as key hot-spot for the development of anti-anthrax vaccines (Figure 1B) (Hoelzgen et al., 2021).

## Conclusions

Despite the successes in clinic for different protein interaction systems involved in diseases, the results of the recent years have highlighted that some PPI are indeed difficult to tackle. Whether a system can be effectively modulated depends on the structural features of the protein-protein complex, and on the possibility to study the system using different biophysical methodologies. Among these, X-ray crystallography and single particle cryo-EM, will continue to be central to successfully assess the structural and mechanistic details of interaction events at atomic resolution, and essential for the development of powerful modulators, be they small molecules, structural peptides, or antibodies. Coupled with further understanding of the nature of identified interactions by genetic and proteomics approaches, the structure determination of PPI will provide a direct path to the development of novel therapeutics targeting these challenging systems. The next future is likely to face the need of further technology innovations in the field. Bifunctional and covalent inhibitors might be fundamentally new ways to target specific PPI systems (Maniaci and Ciulli, 2019).
